# Effects of Dietary Exposure to Zearalenone (ZEN) on Carp (*Cyprinus carpio* L.)

**DOI:** 10.3390/toxins7093465

**Published:** 2015-08-26

**Authors:** Constanze Pietsch, Susanne Kersten, Hana Valenta, Sven Dänicke, Carsten Schulz, Patricia Burkhardt-Holm, Ranka Junge

**Affiliations:** 1 Institute of Natural Resource Sciences (IUNR), Zurich University of Applied Sciences (ZHAW), Gruental, P.O. Box, Waedenswil CH-8820, Switzerland; E-Mail: ranka.junge@zhaw.ch; 2 Man Society Environment, Department of Environmental Sciences, University of Basel, Vesalgasse 1, Basel CH-4051, Switzerland; E-Mail: patricia.holm@unibas.ch; 3 Friedrich-Loeffler-Institute (FLI), Federal Research Institute for Animal Health, Institute of Animal Nutrition, Bundesallee 50, Braunschweig 38116, Germany; E-Mails: susanne.kersten@fli.bund.de (S.K.); hana.valenta@fli.bund.de (H.V.); sven.daenicke@fli.bund.de (S.D.); 4 Christian Albrechts-Universität zu Kiel, Institut für Tierzucht und Tierhaltung, Olshausenstr. 40, Kiel 24098, Germany; E-Mail: cschulz@tierzucht.uni-kiel.de; 5 Gesellschaft für Marine Aquakultur (GMA) mbH, Hafentörn 3, Büsum D-25761, Germany; 6 Department of Biological Sciences, University of Alberta, CW 405 Biological Sciences Building, Edmonton, AB T6G 2E9, Canada

**Keywords:** feed quality, aquaculture, blood cell populations, genotoxicity, estrogenic potential

## Abstract

The mycotoxin zearalenone (ZEN) is frequently contaminating animal feeds including feed used in aquaculture. In the present study, the effects of dietary exposure to ZEN on carp (*Cyprinus carpio* L.) were investigated. ZEN at three different concentrations (low dose: 332 µg kg^−1^, medium dose: 621 µg kg^−1^ and high dose: 797 µg kg^−1^ final feed, respectively) was administered to juvenile carp for four weeks. Additional groups received the mycotoxin for the same time period but were fed with the uncontaminated diet for two more weeks to examine the reversibility of the ZEN effects. No effects on growth were observed during the feeding trial, but effects on haematological parameters occurred. In addition, an influence on white blood cell counts was noted whereby granulocytes and monocytes were affected in fish treated with the medium and high dose ZEN diet. In muscle samples, marginal ZEN and α-zearalenol (α-ZEL) concentrations were detected. Furthermore, the genotoxic potential of ZEN was confirmed by analysing formation of micronuclei in erythrocytes. In contrast to previous reports on other fish species, estrogenic effects measured as vitellogenin concentrations in serum samples were not increased by dietary exposure to ZEN. This is probably due to the fact that ZEN is rapidly metabolized in carp.

## 1. Introduction

The mycotoxin zearalenone (ZEN), which is produced by fungi of the genus *Fusarium*, is increasingly recognized as a frequent contaminant in animal feeding. Mycotoxin contents in fish feeds have rarely been investigated but recent data show that ZEN is highly prevalent in ingredients and complete feedstuffs in aquaculture [1,2,3]. Accordingly, the concentrations of ZEN have been chosen corresponding to specifications in the literature on high ZEN levels (up to 1500 µg kg^−1^) in grains and feedstuff in Europe for the present study [4,5,6,7,8,9].

ZEN and its metabolites have been found to be hepatotoxic [10,11,12], immunotoxic [13,14], and genotoxic in mammals [15,16,17]. Since detrimental effects of mycotoxins may influence growth of fish, the present study aimed to investigate the effects on ZEN on growth performance in carp (*Cyprinus carpio* L.), although a study on Atlantic salmon (*Salmo salar* L.) did not show an effect of ZEN feeding at concentrations of up to 0.77 mg ZEN per kg feed for 15 weeks on the weight gain of this fish species [18]. However, the sensitivity of fish species to mycotoxins is considered to be different due to species-specific and age-specific differences and differences in habitat preferences, physiology and metabolism of toxins [19]. Thus, it is reasonable to investigate growth performance of carp after dietary ZEN exposure.

For the present study, also blood parameters were investigated in carp since a previous study revealed that biochemical parameters in rainbow trout are influenced by ZEN exposure [20]. In addition, ZEN has been assumed to induce suicidal death of erythrocytes in higher vertebrates *via* different mechanisms [21,22,23]. Since also genotoxic effects of ZEN on cells of higher vertebrates have been described [15,16,17], the micronucleus assay (MN assay) has been used [24] to investigate possible chromosomal damage in carp as well. The MN assay is used to detect micronuclei which are formed by whole chromosomes or chromosome fragments that are not reassembled into the nucleus during cell division [25]. This assay is routinely used as index of effects on chromatin because it is easy to perform and sensitive [24]. An increase in the number of micronuclei has also been shown in a mammalian cell line treated with ZEN [26] providing evidence for apoptotic and mutagenic actions of this mycotoxin. A previous study indicated genotoxic effects on permanent fish cells *in vitro* [27], but similar effects *in vivo* have not yet been shown in fish.

Furthermore, ZEN may influence the endocrine system in vertebrates since it is known to contribute to the estrogenic potential in aquatic ecosystems [28,29,30], and has been shown to act as a typical estrogenic agonist showing high estrogenic potencies *via* estrogen receptors including estrogen receptors of rainbow trout [31]. However, quite recently also an anti-androgenic potential of ZEN has been described in a mammalian *in vitro* model [32]. Furthermore, ZEN is commonly metabolized to α-zearalenol (α-ZEL), β-zearalenol (β-ZEL), zearalanone (ZAN), α-zearalanol (α-ZAL) and β-zearalanol (β-ZAL) in mammals and the estrogenic potencies of these major metabolites varies considerably [33,34,35]. Glucuronidation of ZEN and its major metabolites has been shown to reduce their estrogenic potential [36]. The endocrine potential and impact of ZEN and its metabolites on the reproduction in fish have rarely been investigated [37,38,39]. Thus, effects of this substance can be due to its endocrine potential as well as other mechanisms of action such as oxidative stress.

The present study, therefore, aimed to causally determine effects of experimentally ZEN-contaminated diets on growth performance and blood parameters of carp (*Cyprinus carpio* L.) to reveal possible mechanisms of action of this mycotoxin in this fish species since carp is important for aquaculture [40].

## 2. Results and Discussion

### 2.1. Effects on Growth Performance

Contamination of experimental diets containing the low dose of ZEN was comparable to levels that can be found in commercially available feeding stuffs [3]. The two higher concentrations exceed the values that have been found in commercial fish feed up to now but showed lower concentration than the current guidance value by the European Commission of 2 mg kg^−1^ feed [41].

**Table 1:** Zearalenone (ZEN) and α-zearalenol (α-ZEL) concentrations in muscle [ng g^−1^ dry weight]; samples taken from six fish (pooled to two samples) 12 h after the last feeding of fish for four weeks with ZEN-contaminated feeds and after additional two weeks of recovery compared to fish fed the uncontaminated control diet, mean ± standard deviation; n.d. = not detectable.

White Muscle Contamination	Basal feed	Low ZEN	Medium ZEN	High ZEN
After ZEN-treatment				
ZEN [ng g^−1^ dry weight]	n.d.	0.13 ± 0.03	0.22 ± 0.18	0.15 ± 0.07
α-ZEL [ng g^−1^ dry weight]	n.d.	0.11 ± 0.03	0.16 ± 0.11	0.05 ± 0.07
**After Recovery**				
ZEN [ng g^−1^ dry weight]	0.04 ± 0.06	0.03 ± 0.03	0.03 ± 0.02	0.03 ± 0.03
α-ZEL [ng g^−1^ dry weight]	n.d.	n.d.	n.d.	n.d.

After four weeks of experimental feeding, ZEN and α-zearalenol (α-ZEL) have been identified in muscle samples carp (Table 1). Metabolization of ZEN in fish has rarely been described *in vivo* whereby ZEN was metabolized to α-ZEL and β-zearalenol (β-ZEL) in trout liver [42] which implies that similar to mammals the ability to hydroxylate ZEN *via* 3α- and 3β-hydroxysteroid dehydrogenases is present in fish [43]. Nevertheless, ZEN was also found to be metabolized to zearalanone (ZAN), alpha-zearalanol (α-ZAL), beta-zearalanol (β-ZAL), and zearalenol (ZEL) in a mammalian cell line [44,45] which has so far not been observed in fish or fish cells [27,42]. The observed levels for ZEN and α-ZAL are very low in carp muscle tissue, probably due to the fact that fish cells are able to metabolize ZEN rapidly [27].

After four weeks of feeding, two tanks per treatment containing six fish each were sampled, whereas two similarly treated tanks, also containing 12 fish, were fed the uncontaminated diet for further two weeks and were sampled thereafter. At each sampling, growth performance and blood parameters of all fish were analyzed. Accordingly, a significant difference between the initial weights of the fish and the weights at the samplings was observed for all treatment groups (*p* < 0.05) with an exception of the control fish during the first four weeks of experiments. Furthermore, final body weight and length of fish was not influenced by ZEN treatment for four weeks and also not after two additional weeks of recovery (Table 2 and Table 3). Therefore, no differences in condition factors were observed. Furthermore, all fish showed no differences in individual average weight gain per week during the experiments although the fish in the high dose-treated group showed nearly 40% lower values after six weeks of experiments than the fish in the control group which was also accompanied by a higher feed conversion ratio (FCR) in the ZEN-fed group. However, the specific growth rates (SGR) were not significantly influenced by ZEN feeding for four weeks. Weight gain of Atlantic salmon was also not influenced by feeding ZEN concentrations of up to 0.77 mg ZEN per kg feed for 15 weeks [18]. This indicates that growth performance is not a sensitive marker for ZEN effects in fish. However, in the present study, the rather short duration of experimental feeding does not allow making assumptions about chronical effects of ZEN. Therefore, the effects of prolonged ZEN feeding on carp should be investigated in further studies. This might be necessary since higher vertebrates show influences on growth performance due to ZEN feeding. Accordingly, a previous study on male rats showed that ZEN feeding with 1 and 3 mg per kg decreased their weight gain [46] while another study showed negative effects of ZEN feeding on growth of rats of both sexes [47]. In pigs, ZEN feeding not only reduces weight gain but also leads to growth retardation of litter and early embryonic death due to exposure of pregnant gilts and sows to more than 2.8–3 mg per kg ZEN [48]. In contrast, poultry did not show effects on body weight gain after exposure to dietary ZEN concentrations ranging from 10 to 800 mg per kg, and can therefore be considered as being rather insensitive to this mycotoxin compared to other vertebrates [49].

The splenosomatic index was significantly reduced in fish treated with the low dose ZEN diet for four weeks (Table 2) but not in any of the other treatment groups (Table 2 and Table 3). The relative spleen weight in goldfish, *Carassius auratus,* which also belongs to the cyprinids, is known to be sensitive to stress, temperature, increased oxygen demand and hypoxia [50]. It was, furthermore, concluded that the extent of the splenic response depends on the quality and intensity of the imposed stress [50]. Nevertheless, the reason why only the lowest dietary ZEN concentration led to an effect on the splenosomatic index remains obscure. In cyprinids, mainly pronephric and splenic hematopoietic sites are known to contribute to the release of juvenile cell into circulation [51]. Erythropoiesis is also stimulated by temperature, e.g. it is enhanced by diurnal temperature variation and heat shock in goldfish [51,52]. Thus, release of erythrocytes from the splenic reservoir is known to considerably increase abundances of juvenile cells in blood circulation [52]. However, a significant influence on the ratio of red blood cells to leukocytes could not be observed in carp treated with ZEN (Table 4).

**Table 2:** Growth performance and additional sampling parameters of experimental fish after four weeks of zearalenone (ZEN) feeding, *n* = 6 each, with the exception of the feed conversion ratio (FCR) calculations which were conducted per tank resulting in *n* = 2, mean ± standard error of the mean (SEM).

Sampling Parameters	Basal feed	Low ZEN	Medium ZEN	High ZEN
Initial weight [g]	30.0 ± 3.69	29.5 ± 3.45	28.8 ± 2.97	29.3 ± 3.55
Final weight [g]	44.0 ± 7.42	52.1 ± 7.21	41.8 ± 4.02	47.3 ± 5.88
Weight gain [g fish^−1^ week^−1^]	3.7 ± 0.68	5.7 ± 1.10	3.2 ± 0.35	4.7 ± 0.74
Specific growth rate, SGR [% week^−1^]	4.1 ± 0.06	4.2 ± 0.07	4.0 ± 0.04	4.2 ± 0.07
FCR	1.6 ± 0.41	1.0 ± 0.07	1.8 ± 0.28	1.2 ± 0.20
Final total length [cm]	15.1 ± 0.72	14.0 ± 0.75	13.7 ± 0.43	14.4 ± 0.59
Final condition factor	0.02 ± 0.000	0.02 ± 0.000	0.02 ± 0.001	0.02 ± 0.001
Splenosomatic index	0.2 ± 0.01	0.1 ± 0.02 *	0.1 ± 0.03	0.4 ± 0.15
Gonadosomatic index	2.0 ± 0.60	1.1 ± 0.66	1.7 ± 0.52	1.4 ± 0.73
Ratio males/females	5/1	3/3	4/2	3/3

* significantly different means compared with control fish (significance tested with Mann-Whitney U-tests, *p* < 0.05).

**Table 3:** Growth performance and additional sampling parameters of experimental fish after four weeks of zearalenone (ZEN) feeding with additional two weeks of recovery, *n* = 6 each, with the exception of the feed conversion ratio (FCR) calculations which were conducted per tank resulting in *n* = 2, mean ± standard error of the mean (SEM).

Sampling Parameters	Basal feed	Low ZEN	Medium ZEN	High ZEN
Initial weight [g]	26.5 ± 4.10	25.3 ± 2.87	23.0 ± 0.60	22.0 ± 3.48
Final weight [g]	52.3 ± 6.06	46.6 ± 2.51	45.1 ± 4.08	42.9 ± 5.02
Weight gain [g fish^−1^ week^−1^]	4.3 ± 0.76	3.5 ± 0.36	3.7 ± 0.59	3.5 ± 0.35
Specific growth rate, SGR [% week^−1^]	3.5 ± 0.10	3.5 ± 0.07	3.5 ± 0.07	3.5 ± 0.10
FCR	1.4 ± 0.25	1.6 ± 0.06	1.6 ± 0.42	1.7 ± 0.24
Final total length [cm]	14.7 ± 0.51	14.5 ± 0.27	14.2 ± 0.41	13.5 ± 0.58
Final condition factor	0.02 ± 0.000	0.02 ± 0.000	0.02 ± 0.000	0.02 ± 0.001
Splenosomatic index	0.1 ± 0.01	0.1 ± 0.03	0.2 ± 0.03	0.2 ± 0.06
Gonadosomatic index	2.0 ± 1.02	2.2 ± 0.84	1.0 ± 0.33	2.7 ± 1.04
Ratio males/females	3/3	3/3	2/4	3/3

**Table 4:** Differential blood cell counts and micronuclei (MN) occurrence in zearalenone (ZEN)-treated fish and in fish after recovery of two weeks, *n* = 6 per group, mean ± standard error of the mean (SEM); asterisks indicate means that are significantly different from control fish (significance tested with Mann-Whitney U-tests, *p* < 0.05).

Blood cell counts	Basal feed	Low ZEN	Medium ZEN	High ZEN
**ZEN-treated**				
leukocytes [% total blood cells]	3.4 ± 0.20	3.7 ± 0.31	3.4 ± 0.35	3.1 ± 0.32
lymphocytes [% all white blood cells]	60.9 ± 2.06	58.1 ± 3.83	63.3 ± 4.87	60.9 ± 2.42
thrombocytes [% all white blood cells]	31.1 ± 2.92	32.1 ± 2.81	27.6 ± 4.37	30.0 ± 2.89
monocytes [% all white blood cells]	4.8 ± 0.49	4.2 ± 0.61	2.9 ± 0.40 *	3.1 ± 0.30 *
granulocytes [% all white blood cells]	3.2 ± 0.78	5.7 ± 1.69	6.2 ± 1.34 *	6.1 ± 0.85 *
MN in erythrocytes [per 1000 cells]	12.9 ± 5.13	43.5 ± 9.66 *	29.9 ± 2.92 *	36.54 ± 7.5 *
**Recovery**				
leukocytes [% total blood cells]	3.6 ± 0.54	2.8 ± 0.32	4.9 ± 0.50	2.7 ± 0.36
lymphocytes [% all white blood cells]	59.6 ± 3.30	59.3 ± 1.56	65.6 ± 3.80	64.0 ± 4.92
thrombocytes [% all white blood cells]	34.3 ± 2.77	30.2 ± 2.41	29.8 ± 3.39	29.0 ± 4.95
monocytes [% all white blood cells]	2.6 ± 0.49	3.8 ± 0.97	2.8 ± 0.44	2.5 ± 0.40
granulocytes [% all white blood cells]	3.4 ± 0.50	6.7 ± 1.43	1.8 ± 0.34	4.7 ± 1.01

* significantly different means compared with control fish (significance tested with Mann-Whitney U-tests, *p* < 0.05).

### 2.2. Hematology

Haemoglobin and haematocrit were not significantly influenced by ZEN with the exception of fish treated with the medium dose feed after two weeks of recovery showing significantly lower mean haemoglobin levels than control fish (Table 5). This may be due to eryptosis which can be triggered by several intracellular and extracellular signals [53]. On the one hand, ZEN-treated rats showed reduced haemoglobin and haematocrit after injection with the mycotoxin for 3–24 h [21] which was assumed to be due to cytopathic effects of ZEN [22]. On the other hand, another study provided evidence for calcium-triggered cell death of erythrocytes due to ZEN exposure [23]. Last but not least, oxidative stress caused by ZEN exposure is involved in cytotoxicity of ZEN in fish cells [27]. Influences of hypoxia and temperature on haematological parameters can be excluded, since these are known to increase haemoglobin concentrations in cyprinids [50], and temperature and oxygen levels were within optimal range for all fish within the experiment. In contrast, intoxication with chemicals such as the insecticide endosulfan led to reduced haemoglobin levels in carp indicating a pathological condition in the animals [54]. This could also be true for the ZEN-treated carp. However, fish possess different types of anodal and cathodal haemoglobins, and variations of temperature, oxygen availability and other factors are associated with changes in the abundance of these haemoglobin types [55]. Therefore, the exact reason for the observed effects on the haemoglobin concentrations in the group fed the medium dose ZEN diet can be multifactorial and remains unknown.

In the present study, effects of ZEN treatment on serum vitellogenin values have not been observed (Table 5). Thus, the potential effects on reproduction as have been observed in other fish species [37,38,39] after exposure to 2 ng ZEN per L as well as concentrations of up to 5000 ng ZEN per L *via* the surrounding water could not be confirmed in carp treated with ZEN *via* the diet for four weeks which might also indicate that the route of exposure is important for ZEN effects on fish. Consequently, the exact effects of ZEN on the endocrine system, especially in fishes, still need to be investigated more in detail. Besides estrogenic effects, a recent study also showed anti-androgenic potential of ZEN in a mammalian *in vitro* model system [32]. In addition, glucuronidation of ZEN commonly occurs in vertebrate cells including fish cells [27,56], and is known to considerably reduce estrogenic acitivy of ZEN and its major metabolites [36]. This probably also had a strong influence on ZEN action in carp.

**Table 5:** Haematological parameters of zearalenone (ZEN)-treated fish and in fish after recovery of two weeks, *n* = 6 per group, mean ± standard error of the mean (SEM).

Haematological Parameters	Basal feed	Low ZEN	Medium ZEN	High ZEN
**ZEN-treated**				
Haematocrit [%]	28.4 ± 1.51	32.6 ± 1.85	28.7 ± 0.75	32.9 ± 2.33
Haemoglobin [mg dL^−1^]	7.9 ± 0.46	9.1 ± 0.68	8.7 ± 0.50	8.9 ± 0.54
Vitellogenin [ng mL^−1^]	104 ± 3.5	112 ± 6.8	103 ± 1.9	108 ± 3.1
**Recovery**				
Haematocrit [%]	29.1 ± 0.97	29.3 ± 0.82	26.9 ± 0.79	30.5 ± 1.25
Haemoglobin [mg dL^−1^]	8.5 ± 0.24	8.4 ± 0.24	7.6 ± 0.28 *	8.4 ± 0.35
Vitellogenin [ng mL^−1^]	102 ± 1.4	103 ± 3.1	110 ± 4.4	103 ± 1.7

* significantly different means compared with control fish (significance tested with Mann-Whitney U-tests, *p* < 0.05).

Differential blood cell counts revealed significantly more granulocytes and less monocytes in fish treated with the medium and high dose ZEN diet. Different effects on leukocyte populations that lead to effects on cellular immune responses have already been suggested for ZEN-fed sheep [57]. ZEN also exhibited cytopathic effects on isolated peripheral blood mononuclear cells of humans and 30 μg mL^−1^ ZEN entirely inhibited phytohemagglutinin- and pokeweed mitogen-stimulated proliferation of T and B lymphocytes [22]. Since granuocytes and monocytes are important immune effector cells, this indicates that profound immune functions of carp are influenced by ZEN treatment. The effect of ZEN on innate immune responses of carp has been investigated in parallel [58]. These closer examinations of immune functions revealed that immune responses are frequently increased after exposure of carp to low ZEN concentrations and reduced after exposure to high ZEN concentrations. Immunological alterations due to ZEN application have also been observed in the intestine, in thymocytes and splenocytes of rodents [59,60]. The effects of ZEN on immune cells have shown to be related to altered cytokine expression in chicken, swine and rats [61,62,63,64,65,66]. Whether the effects of ZEN on immune function in carp also involve altered cytokine expression remains to be investigated.

Despite the effect on granulocyte and monocyte numbers, the total amount of leukocytes in the blood remained unchanged. After two weeks of recovery, the composition of the white cell population was comparable in all treatment groups.

ZEN has already been found to be genotoxic in fish cells *in vitro* [27]. Similarly, MN numbers were increased by *in vivo* treatment with ZEN in all feeding groups in the present study (Table 4). Although the percentage of MN was higher than observed in carp erythrocytes after microcystin treatment [67], it was comparable to the levels observed in ZEN-treated mice [15]. This is in line with the increasing concern about possible carcinogenic actions of ZEN in vertebrates [46,68], and may be problematic for fish in aquaculture since the ZEN concentrations used in the experimental diets in this study were far below the maximum allowable levels in complete feedstuffs recommended by the European Commission [41].

## 3. Experimental Section

### 3.1. Chemicals

All chemicals were obtained from Sigma-Aldrich (Buchs, Switzerland), unless indicated otherwise.

### 3.2. Preparation of Feeds and Husbandry

Experimental diet composition was chosen without cereals in order to exclude contamination with cereal-based *Fusarium* toxins including ZEN. Fish meal (VFC GmbH, Cuxhaven, Germany), blood meal (Euroduna-Technologies GmbH, Barmstedt, Germany), casein, dextrose, and potato starch were used for feed preparation (at 30%, 12.5%, 12.0%, 13.0%, and 21.1%, respectively). Vitamins and minerals from a vitamin and mineral mix by Spezialfutter Neuruppin (Neuruppin, Germany), Germany-VM BM 55/13 No. 7318 containing Vitamin A 12000 I.E; Vitamin D3 1600 I.E; Vitamin E 160 mg; Vitamin K3 6.4 mg; Vitamin B1 12 mg; Vitamin B2 16 mg; Vitamin B6 12 mg Vitamin B12 26.4 μg; Nicotinic acid 120 mg; Biotin 800 μg; Folic acid 4.8 mg; Pantothenic acid 40 mg, Inositol 240 mg; Vitamin C 160 mg; Antioxidants (BHT) 120 mg; Iron 100 mg; Zink 24 mg; Manganese 16 mg; Cobalt 0.8 mg; Iodine 1.6 mg; Selenium 0.08 mg) made up 1% of the final feeds. The vitamins and minerals were added to diets to meet the dietary requirements of carp [69].

All ingredients were mixed thoroughly. Zearalenone (ZEN, dissolved in ethanol; purity > 99%, obtained from Sigma-Alrich, Buchs, Switzerland, lot-no. 041M4054V) was added to the fish oil (VFC GmbH, Cuxhaven, Germany; accounting for 10.4% of the final feed) at three different concentrations (low dose: 332 µg kg^−1^, medium dose: 621 µg kg^−1^ and high dose: 797 µg kg^−1^ final feed, respectively) prior to addition of the other ingredients, so that the final ethanol content in all experimental diets accounted for less than 0.015% (*v/w*). Diets were manufactured to 4 mm pellets in a pelletizer (L 14-175, Amandus Kahl, Reinbek, Germany) and allowed to cool down to room temperature for two hours before storage at 4 °C until use. The composition of the diets was analysed by using standard methods. Accordingly, the experimental diets were analysed for dry matter (DM) (105 °C, until constant weight), crude ash (550 °C, 2 h), crude fat (Soxtec HT6, Tecator, Höganäs, Sweden) and crude protein content (N x 6.25; Kjeltec Auto System, Tecator, Höganäs, Sweden). Nitrogen-free extract and fibres (NFE) were summarized as shown in Equation (1).

% NFE = 100 – (% protein + % fat + % ash)
(1)

The diets were formulated to be isonitrogenous (46.65 ± 0.26% crude protein, mean ± SD) and isocaloric (22.57 ± 0.11 MJ kg^−1^ dry matter, mean ± SD) and the exact nutritional composition can be seen in the table in the Supplementary Materials.

The experimental conditions were monitored by regular measurements of water parameters which showed average values of 7.3 ± 0.2 mg L^−1^ for dissolved oxygen, a conductivity of 210 ± 2 μS m^−1^ and a pH value of 8.1 ± 0.0 (mean ± SD) for all tanks during the entire experimental phase.

### 3.3. Analysis of ZEN in Experimental Diets and ZEN and Its Metabolites in Muscle Samples

ZEN in feed was determined by HPLC with fluorescence detection after a clean-up with IAC (immunoaffinity column, ZearalaTest, Vicam, Klaus Ruttmann, Hamburg, Germany) according to the method of the “Verband Deutscher Landwirtschaftlicher Untersuchungs- und Forschungsanstalten” [70] as has been described previously [71]. The detection limit was 2 μg kg^−1^ and mean recovery was approximately 79%.

In muscle, ZEN and the metabolites α-zearalenol (α-ZEL), β-zearalenol (β-ZEL), zearalanone (ZAN), α-zearalanol (α-ZAL) and β-zearalanol (β-ZAL) were analysed with LC-MS/MS after clean-up with IAC. In detail, pooled muscle samples from three fish per treatment were freeze-dried and stored at −20 °C until analyses. For analysis, 2.4 g of the freeze-dried sample was mixed with 7 mL double-distilled water, followed by addition of 5 mL sodium acetate buffer (pH 5.5) and incubation with 200 µL β-glucuronidase solution (Sigma G 0876, 100,000 U/mL) at 37 °C overnight. Afterwards, samples were extracted with 60 mL acetonitrile for 1 h with continuous agitation (240 U min^−1^) and filtered using fluted filters. Prior to IAC clean-up, the extracts were pre-cleaned according to a modified procedure for the determination of DON and de-epoxy DON in animal tissues [72] whereby charcoal was omitted because of absorption of ZEN. In brief, 40 mL of the obtained filtrate were mixed with 40 mL petrol ether for 30 s and the acetonitrile-water-phase was mixed with 3 g of a mixture of aluminium oxide and Celite (5:3; *w:w*) followed by 10 min shaking. After 5 min of sedimentation, samples were filtered using fluted filters. A portion of 25 mL of the filtrate was mixed with 25 mL ethanol and evaporated using a rotation vacuum evaporator at 60 °C, and remaining solvent residues were removed under gentle nitrogen stream. The resulting samples were solubilized in 3 mL acetonitrile in an ultrasonic bath followed by 17 mL phosphate buffered saline (pH 7.3) and the solution was filtered through a glass fibre filter when necessary. Subsequently, the solution was cleaned up with IAC (ZearalaTest, Vicam; distributed by Klaus Ruttmann GmbH, Hamburg, Germany) according to the procedure for feed as mentioned above. ZEN and the metabolites were determined with LC-ESI-MS/MS in negative mode according to a previous study [71]. The limits of quantification (LOQ) for the six toxins were in the range of 0.02 ng g^−1^ (ZEN) to 0.14 ng g^−1^ (β-ZEL), all related to freeze-dried tissue. This corresponds to approximately 0.005–0.035 ng g^−1^ in fresh tissue. The mean recovery of the six substances was in the range of 78%–97%. The results from the ZEN and α-ZEL analysis in muscle samples are shown in Table 1.

### 3.4. Experimental Feeding Design

Carp were raised from eggs in the experimental facilities and used for the experiments at 12–16 cm in length. Fish were kept at a 16 h light/8 h dark photoperiod at 24.9 ± 0.4 °C (mean ± SD) in a flow through system. Fish were acclimated for at least three weeks to the experimental tanks where all animals received the uncontaminated experimental diet. The prepared pellets (4 mm in diameter) were given at 3% of body mass as two meals every day to juvenile carp which were separated into four different feeding groups (control, low dose, medium dose, and high dose). All experimental diets were taken-up without refusal. Each feeding group included four tanks of 54 L containing six fish each. The flow through was adjusted to approximately 6 L fresh water per h for each tank. Water temperature, pH (WTW pH 315i, Wissenschaftlich-Technische Werkstätten GmbH, Weilheim, Germany), conductivity (WTW cond 315i, Weilheim, Germany) and dissolved oxygen (WTW Oxi 330i, Weilheim, Germany) were recorded for each tank at least three times a week. Every tank was cleaned at least every second day. Fish were fed the contaminated diets for four weeks while the control groups received the uncontaminated feed. Uptake of feed was observed in all groups within less than 30 min after offering the experimental diets. Feed amounts per tank were adjusted to the increased weight on a weekly basis. After four weeks of experimental feeding, two tanks per feeding group were sampled. The remaining two tanks per feeding group were fed the uncontaminated diet for additional two weeks before the final sampling to investigate possible recovery from ZEN feeding. Sampling of fish included blood sampling, recording of weight and length, as well as sampling of individual organs. Specific growth rates (SGR, expressed as % per day), feed conversion ratios (FCR) and condition factors (CF) were calculated according to Equations (2), (3) and (4), respectively. All experimental procedures have been approved by the Cantonal veterinarian authorities of Basel-Stadt (Switzerland) under the permission number 2410.

(2) $$SGR=\frac{\ln final\;weight-\;\ln initial\;weight\;}{days\;of\;experiment}\;\times 100$$

(3) $$FCR=\frac{used\;feed\;amount}{\left(final\;weight-initial\;weight\right)}$$

(4) $$CF=\frac{weight}{length^{3}}$$

### 3.5. Determination of Haematological Parameters

From individual fish, blood was drawn from the caudal vein using heparinized syringes immediately after removal from each tank. For haemoglobin determination according to the Drabkin method [73], 5 µL of freshly drawn blood was added to Drabkin’s solution containing 30% Brij^®^ 35 and absorptions were read at 540 nm (Infinite M200, Tecan Group Ltd., Männedorf, Switzerland). A standard curve was prepared using human lyophilized haemoglobin. Haematocrit was assessed using heparinized glass capillary tubes (Huber & Co. AG, Reinach, Switzerland) in duplicate which were analysed after centrifugation at 3000 rpm for 10 min (Haematokrit Typ 2010, Hettich Zentrifugen, Tuttlingen, Germany).

### 3.6. Determination of Differential Blood Cell Counts and Micronuclei Counts

From each fish, two blood smears prepared from freshly drawn blood samples on glass slides were allowed to air-dry. Thereafter, the slides were stained with Wright-Giemsa stain (Sigma-Aldrich, Buchs, Switzerland) according to the manufacturer’s protocol and 10 pictures were taken randomly at a 400× magnification from each slide (using a Nikon Eclipse E400 equipped with a Nikon Digital Camera DXM1200F from Nikon AG Instruments, Egg (ZH), Switzerland). All blood cells from each picture were counted so that an average number of 7794 cells per fish (mean; maximum number: 9908 cells; minimum number: 5747 cells) were used in total for calculating differential blood cell counts from individual fish. For the micronuclei assay small, non-refractive, circular or ovoid chromatin bodies classified as micronuclei were counted in erythrocytes when they displayed the same staining and focusing pattern as the main nuclei [74].

### 3.7. Analysis of Plasma Vitellogenin

After preparation of blood samples, plasma was obtained by centrifugation at 3000× *g* for 20 min at 4 °C (Centrifuge 5415R, Eppendorf, Basel, Switzerland). For analyses the Carp Vitellogenin ELISA kit from Cusabio (Chemie Brunschwig AG, Basel, Switzerland) was used according to the manufacturer’s protocol.

### 3.8. Statistics

Data are presented as the mean ± standard error of the mean (SEM) unless indicated otherwise. Effects of treatments were determined by comparison of treatment groups to controls using non-parametrical Kruskal-Wallis tests followed by Mann-Whitney U-tests (SPSS 9.0 for Windows; SPSS Inc, Chicago IL, USA, 1999). A significance value (*p*) of <0.05 was accepted as being statistically significant.

## 4. Conclusions

The alterations in ZEN-treated carp suggest that the fish may face metabolic as well as immunological and genotoxic influences. This raises concern about the recommended maximum allowable levels for ZEN in feedstuffs. The present study allows new insights into the mechanisms of action of ZEN, although all the effects that are induced are still not completely understood. Thus, the specific biological and molecular action of ZEN on cell functions *in vivo* is still unclear and needs to be elucidated in further studies.

## Supplementary Materials

Supplementary materials can be accessed at: http://www.mdpi.com/2072-6651/7/9/3465/s1.

## References

[1] Måge A., Julshamn K., Lunestad B.T. (2009). Overvåkningsprogram for förvarer til fisk og andre akvatiske dyr e Årsrapport 2008 og 2009.

[2] Santos G.A., Rodrigues I., Naehrer K., Encarnacao P. (2010). Mycotoxins in aquaculture: occurrence in feed components and impact on animal performance. Aquac. Eur..

[3] Pietsch C., Kersten S., Burkhardt-Holm P., Valenta H., Dänicke S. (2013). Occurrence of deoxynivalenol and zearalenone in commercial fish feed—An initial study. Toxins.

[4] Coppock R.W., Mostrom M.S., Sparling C.G., Jacobsen B., Ross S.C. (1990). Apparent zearalenone intoxication in a dairy herd from feeding spoiled acid treated corn. Vet. Hum. Toxicol..

[5] Veldman A., Borggreve G.J., Mulders E.J., Van de Lagemaat D. (1992). Occurence of the mycotoxins ochratoxin A, zearalenone and deoxynivalenol in feed components. Food Add. Contam..

[6] Charmley L.L., Rosenberg A., Trenholm H.L., Miller J.D., Trenholm H.L. (1994). Factors responsible for economic losses due to Fusarium mycotoxin contamination of grains, foods and feedstuffs. Mycotoxins in Grain-Compounds Other than Aflatoxin.

[7] Whitlow L.W., Hagler W.M. (2005). Mycotoxins in feeds. Feedstuffs.

[8] Mankeviciene A., Butkute B., Dabkevicius Z., Suproniene S. (2007). Fusarium mycotoxins in lithuanian cereals from the 2004e05 harvests. Ann. Agric. Environ. Med..

[9] Driehuis F., Spanjer M.C., Scholten J.M., Te Giffel M.C. (2008). Occurrence of mycotoxins in feedstuffs of dairy cows and estimation of total dietary intakes. J. Dairy Sci..

[10] Maaroufi K., Chekir L., Creppy E.E., Ellouz F., Bacha H. (1996). Zearalenone induces modifications of haematological and biochemical parameters in rats. Toxicon.

[11] Obremski K., Zielonka L., Zaluska G., Zwierzchowski W., Pirus K., Gajecki M. The influence of low doses of zearalenone on liver enzyme activities in gilts. Proceedings of the X Conference “Microscopic Fungi—Plant Pathogens and their Metabolites”.

[12] Conkova E., Laciakova A., Pastorova B., Seidel H., Kovac G. (2001). The effect of zearalenone on some enzymatic parameters in rabbits. Toxicol. Lett..

[13] Marin L., Murtha J., Dong W., Pestka J.J. (1996). Effects of mycotoxins on cytokine production and proliferation in EL-4 thymoma cells. J. Toxicol. Environ. Health.

[14] Berek L., Petri I.B., Mesterhazy A., Teren J., Molnar J. (2001). Effects of mycotoxins on human immune functions *in vitro*. Toxicol. In Vitro.

[15] Ouanes Z., Abid S., Ayed I., Anane R., Mobio T., Creppy E., Bacha H. (2003). Induction of micronuclei by zearalenone in Vero monkey kidney cells and in bone marrow cells of mice: protective effect of vitamine E. Mutat. Res..

[16] Abid-Essefi S., Ouanes Z., Hassen W., Baudrimont I., Creppy E.E., Bacha H. (2004). Cytotoxicity, inhibition of DNA and protein syntheses and oxidative damage in cultured cells exposed to zearalenone. Toxicol. In Vitro.

[17] Lioi M.B., Santoro A., Barbieri R., Salzano S., Ursini M.V. (2004). Ochratoxin and zearalenone: A comparative study on genotoxic effects and cell death induced in bovine lymphocytes. Mutat. Res..

[18] Döll S., Valenta H., Baardsen G., Möller P., Koppe W., Stubhaug I., Dänicke S. Effects of increasing concentrations of deoxynivalenol, zearalenone and ochratoxin A in diets for Atlantic salmon (*Salmo salar*) on performance, health and toxin residues. Proceedings of the Abstracts of the 33rd Mycotoxin Workshop.

[19] Pietsch C., Pietsch C., Hirsch P.E. (2015). Impact of natural toxins on carp. Biology and Ecology of Carp.

[20] Woźny M., Brzuzan P., Gusiatin M., Jakimiuk E., Dobosz S., Kuźmiński H. (2012). Influence of zearalenone on selected biochemical parameters in juvenile rainbow trout (*Oncorynchus mykiss*). Polish J. Vet. Sci..

[21] Chattopadhyay P., Upadhyay A., Agnihotri A., Karmakar S., Ghoyary D., Veer V. (2013). Comparative hematoxicity of Fusirium mycotoxin in experimental Sprague-Dawley rats. Toxicol. Int..

[22] Vlata Z., Porichis F., Tzanakakis G., Tsatsakis A., Krambovitis E. (2006). A study of zearalenone cytotoxicity on human peripheral blood mononuclear cells. Toxicol Lett..

[23] Jilani K., Lang F. (2013). Ca^2+^-dependent suicidal erythrocyte death following zearalenone exposure. Arch. Toxicol..

[24] Fenech M., Chang W.P., Kirsch V.M., Holland N., Bonassi S., Zeiger E. (2003). HUMN project: detailed description of the scoring criteria for the cytokinesis-block micronucleus assay using isolated human lymphocyte cultures. Mutat. Res..

[25] Heddle J.A., Cimino M.C., Hayashi M., Romagna F., Shelby M.D., Tucker J.D., Vanparys P., MacGregor J.T. (1991). Micronuclei as an index of cytogenetic damage: past, present, and future. Environ. Mol. Mutagen..

[26] Ayed-Boussema I., Ouanes Z., Bacha H., Abid S. (2007). Toxicities induced in cultured cells exposed to zearalenone: Apoptosis or mutagenesis?. J. Biochem. Mol. Toxicol..

[27] Pietsch C., Noser J., Wettstein F.E., Burkhardt-Holm P. (2014). Unravelling the mechanisms involved in zearalenone-mediated toxicity in permanent fish cell cultures. Toxicon.

[28] Bucheli T.D., Wettstein F.E., Hartmann N., Erbs M., Vogelsang S., Forrer H.-R., Schwarzenbach R.P. (2008). *Fusarium* mycotoxins: Overlooked aquatic micropollutants. J. Agric. Food Chem..

[29] Hoerger C., Schenzel J., Strobel B., Bucheli T. (2009). Analysis of selected phytotoxins and mycotoxins in environmental samples. Anal. Bioanal. Chem..

[30] Hartmann N., Erbs M., Forrer H.-R., Vogelsang S., Wettstein F.E., Schwarzenbach R.P., Bucheli T.D. (2008). Occurrence of zearalenone on *Fusarium graminearum* infected wheat and maize field in crop organs, soil, and drainage water. Environ. Sci. Technol..

[31] Bucheli T.D., Erbs M., Hartmann N., Vogelsang S., Wettstein F.E., Forrer H.R. (2005). Estrogenic mycotoxins in the environment. Mitt. Lebensm. Hyg..

[32] Molina-Molina J.-M., Real M., Jimenez-Diaz I., Belhassen H., Hedhili A., Tornè P., Fernàndez M.F., Olea N. (2014). Assessment of estrogenic and anti-androgenic activities of the mycotoxin zearalenone and its metabolites using *in vitro* receptor-specific bioassays. Food Chem. Toxicol..

[33] Benzoni E., Minervini F., Giannoccaro A., Fornelli F., Vigo D., Visconti A. (2008). Influence of *in vitro* exposure to mycotoxin zearalenone and its derivatives on swine sperm quality. Reprod. Toxicol..

[34] De Andrés F., Zougagh M., Castañeda G., Ríos A. (2008). Determination of zearalenone and its metabolites in urine samples by liquid chromatography with electrochemical detection using a carbon nanotube-modified electrode. J. Chromatogr. A.

[35] Filannino A., Stout T., Gadella B., Sostaric E., Pizzi F., Colenbrander B., Dell’Aquila M., Minervini F. (2011). Dose-response effects of estrogenic mycotoxins (zearalenone, alpha- and beta-zearalenol) on motility, hyperactivation and the acrosome reaction of stallion sperm. Reprod. Biol. Endocrinol..

[36] Frizzell C., Uhlig S., Miles C.O., Verhaegen S., Elliott C.T., Eriksen G.S., Sorlie M., Ropstad E., Connolly L. (2015). Biotransformation of zearalenone and zearalenols to their major glucuronide metabolites reduces estrogenic activity. Toxicol. In Vitro.

[37] Johns S.M., Denslow N.D., Kane M.D., Watanabe K.H., Orlando E.F., Sepulveda M.S. (2009). Effects of estrogens and antiestrogens on gene expression of fathead minnow (*Pimephales promelas*) early life stages. Environ. Toxicol..

[38] Schwartz P., Thorpe K.L., Bucheli T.D., Wettstein F.E., Burkhardt-Holm P. (2010). Short-term exposure to the environmentally relevant estrogenic mycotoxin zearalenone impairs reproduction in fish. Sci. Tot. Environ..

[39] Bakos K., Kovács R., Staszny Á., Sipos D.K., Urbányi B., Müller F., Csenki Z., Kovács B. (2013). Developmental toxicity and estrogenic potency of zearalenone in zebrafish (*Danio rerio*). Aquat. Toxicol..

[40] FAO (2012). The State of World Fisheries and Aquaculture.

[41] European Commission (2006). Commission Recommendation (2006/576/EC) of 17 August 2006 on the presence of deoxynivalenol, zearalenone, ochratoxin A, T-2 and HT-2 and fumonisins in products intended for animal feeding. Off. J. Eur. Union.

[42] Lagana A., Faberi A., Fago G., Marino A., Pastorini E., Samperi R. (2004). Application of an innovative matrix solid-phase dispersionesolidphase extractioneliquid chromatographyetandem mass spectrometry analytical methodology to the study of the metabolism of the estrogenic mycotoxin zearalenone in rainbow trout liver and muscular tissue. Int. J. Environ. Anal. Chem..

[43] Olsen M., Pettersson H., Kiessling K.H. (1981). Reduction of zearalenone to zearalenol in female rat liver by 3 alpha-hydroxysteroid dehydrogenase. Acta Pharmacol. Toxicol..

[44] Schaut A., De Saeger S., Sergent T., Schneider Y.-J., Larondelle Y., Pussemier L., Van Peteghem C. (2008). Study of the gastrointestinal biotransformation of zearalenone in a Caco-2 cell culture system with liquid chromatographic methods. J. Appl. Toxicol..

[45] Videmann B., Mazallon M., Tep J., Lecoeur S. (2008). Metabolism and transfer of the mycotoxin zearalenone in human intestinal Caco-2 cells. Food Chem. Toxicol..

[46] Becci P.J., Voss K.A., Hess F.G., Gallo M.A., Parent R.A., Stevens K.R., Taylor J.M. (1982). Long-term carcinogenicity and toxicity study of zearalenone in the rat. J. Appl. Toxicol..

[47] Kiessling K.H. (1982). The effect of zearalenone on growth rate, organ weight and muscle fibre composition in growing rats. Acta Pharmacol. Toxicol. (Copenh).

[48] Kanora A., Maes D. (2009). The role of mycotoxins in pig reproduction: a review. Vet. Med..

[49] Allen N.K., Mirocha C.J., Weaver G., Aakhus-Allen S., Bates F. (1981). Effects of dietary zearalenone on finishing broiler chickens and young turkey poults. Poultry Sci..

[50] Houston A.H., Roberts W.C., Kennington J.A. (1996). Hematological response in fish: pronephric and splenic involvements in the goldfish, *Carassius auratus* L.. Fish Physiol. Biochem..

[51] Houston A.H., Murad A. (1992). Erythrodynamics in goldfish, *Carassius auratus* L.: Temperature effects. Physiol. Zool..

[52] Fange R., Nilsson S., Holgren S. (1986). Physiology of hemopoiesis. Fish physiology: recent advances.

[53] Lang E., Lang F. (2015). Triggers, inhibitors, mechanisms, and significance of eryptosis: The suicidal erythrocyte death. BioMed Res. Int..

[54] Al-Rudainy A.J., Kadhim M.H. (2012). Hematological and neurotoxic effects of endosulfan pesticide on common carp *Cyprinus carpio*. The Iraqi J. Vet. Med..

[55] Tun N., Houston A.H. (1986). Temperature, oxygen, photoperiod and the hemoglobin system of the rainbow trout, Salmo gairdneri. Can. J. Zool..

[56] Warth B., Sulyok M., Berthiller F., Schuhmacher R., Krska R. (2013). New insights into the human metabolism of the Fusarium mycotoxins deoxynivalenol and zearalenone. Toxicol. Lett..

[57] Kostro K., Gajecka M., Lisiecka U., Majer-Dziedzic B., Obremski K., Zielonka L., Gajecki M. (2011). Subpopulation of lymphocytes CD^4+^ and CD^8+^ in peripheral blood of sheep with zearalenone mycotoxicosis. Bull. Vet. Inst. Pulawy.

[58] Pietsch C., Junge R., Burkhardt-Holm P. (2015). Immunomodulation by zearalenone (ZEN) in carp (*Cyprinus carpio* L.). BioMed Research Int..

[59] Abbès S., Ben Salah-Abbès J., Sharafi H., Noghabi K.A., Oueslati R. (2012). Interaction of *Lactobacillus plantarum* MON03 with Tunisian Montmorillonite clay and ability of the composite to immobilize Zearalenone *in vitro* and counteract immunotoxicity *in vivo*. Immunopharmacol. Immunotoxicol..

[60] Liu M., Gao R., Meng Q., Zhang Y., Bi C., Shan A. (2014). Toxic effects of maternal zearalenone exposure on intestinal oxidative stress, barrier function, immunological and morphological changes in rats. PLoS ONE.

[61] Marin D.E., Taranu I., Burlacu R., Tudor D.S. (2010). Effects of zearalenone and its derivatives on the innate immune response of swine. Toxicon.

[62] Marin D.E., Taranu I., Burlacu R., Manda G., Motiu M., Neagoe I., Dragomir C., Stancu M., Calin L. (2011). Effects of zearalenone and its derivatives on porcine immune response. Toxicol. In Vitro.

[63] Wang Y.C., Deng J.L., Xu S.W., Peng X., Zuo Z.C., Cui H.M., Wang Y., Ren Z.H. (2012). Effects of zearalenone on IL-2, IL-6, and IFN-gamma mRNA levels in the splenic lymphocytes of chickens. Sci. World J..

[64] Choi B.-K., Cho J.-H., Jeong S.-H., Shin H.-S., Son S.-W., Yeo Y.-K., Kang H.-G. (2012). Zearalenone affects immune-related parameters in lymphoid organs and serum of rats vaccinated with porcine parvovirus vaccine. Toxicol. Res..

[65] Pistol G.C., Braicu C., Motiu M., Gras M.A., Marin D.E., Stancu M., Calin L., Israel-Roming F., Berindan-Neagoe I., Taranu I. (2015). Zearalenone mycotoxin affects immune mediators, MAPK signalling molecules, nuclear receptors and genome-wide gene expression in pig spleen. PLoS ONE.

[66] Obremski K. (2014). Changes in Th1 and Th2 cytokine concentrations in ileal Peyer’s patches in gilts exposed to zearalenone. Polish J. Vet. Sci..

[67] Wu Q., Li M., Gao X., Giesy J.P., Cui Y., Yang L., Kong Z. (2011). Genotoxicity of crude extracts of cyanobacteria from Taihu Lake on carp (*Cyprinus carpio*). Ecotoxicol..

[68] Duca R.-C., Bravin F., Delaforge M. (2006). Study of zearalenone contaminated feedstuffs on the detoxification enzymes. Archiva Zootechnica.

[69] National Research Council (NRC) (1993). Nutrient Requirements of Fish.

[70] (2006). VDLUFA-Methodenbuch III. 6. Ergänzung 2006, Zearalenon 16.9.2.

[71] Brezina U., Valenta H., Rempe I., Kersten S., Humpf H.-U., Dänicke S. (2014). Development of a liquid chromatography tandem mass spectrometry method for the simultaneous determination of zearalenone, deoxynivalenol and their metabolites in pig serum. Mycotox. Res..

[72] Valenta H., Dänicke S., Döll S. (2003). Analysis of deoxynivalenol and de-epoxy-deoxynivalenol in animal tissues by liquid chromatography after clean-up with an immunoaffinity column. Mycotox. Res..

[73] Drabkin D.L., Austin J.H. (1935). Spectrophotometric studies. II. Preparations from washed blood cells; nitric oxide hemoglobin and sulfhemoglobin. J. Biol. Chem..

[74] Al-Sabti K., Metcalfe C.D. (1995). Fish micronuclei for assessing genotoxicity in water. Mutat. Res..

