# Supplementary material for: Effects of Dietary Exposure to Zearalenone (ZEN) on Carp (Cyprinus carpio L.)
Source: Toxins (Basel). 2015 Aug 26;7(9):3465–80. doi: 10.3390/toxins7093465 (PMC4591655; doi:10.3390/toxins7093465)
Supplement: Supplementary file 1 [file toxins-07-03465-s001.pdf]

Supplementary Information

Table S1. Composition of the experimental fish feeds, the values are given as means ± SD of two independent determinations of the same feed batch, NFE = nitrogen-free extract.

Feed Composition	Basal feed	Low ZEN	Medium ZEN	High ZEN
Dry matter [% wet matter]	89.27 ± 0.70	89.80 ± 0.04	89.74 ± 0.06	89.88 ± 0.06
Crude protein [% dry matter]	46.99 ± 0.41	46.63 ± 0.32	46.35 ± 0.34	46.62 ± 0.21
Crude lipid [% dry matter]	13.64 ± 0.22	13.99 ± 0.62	14.62 ± 0.11	13.38 ± 0.06
Crude ash [% dry matter]	6.98 ± 0.00	6.95 ± 0.11	6.83 ± 0.03	7.02 ± 0.01
NFE [% dry matter]	32.66 ± 0.63	32.43 ± 0.41	32.20 ± 0.26	32.99 ± 0.16
Gross energy [MJ kg^{-1} dry matter]	22.66 ± 0.12	22.65 ± 0.04	22.56 ± 0.08	22.42 ± 0.04
